# Supplementary material for: Reduced Cell Adhesion on LightPLAS-Coated Implant Surfaces in a Three-Dimensional Bioreactor System
Source: Int J Mol Sci. 2023 Jul 18;24(14):11608. doi: 10.3390/ijms241411608 (PMC10380481; doi:10.3390/ijms241411608)
Supplement: Supplementary file 1 [file ijms-24-11608-s001.zip › ijms-2453697-supplementary.pdf]

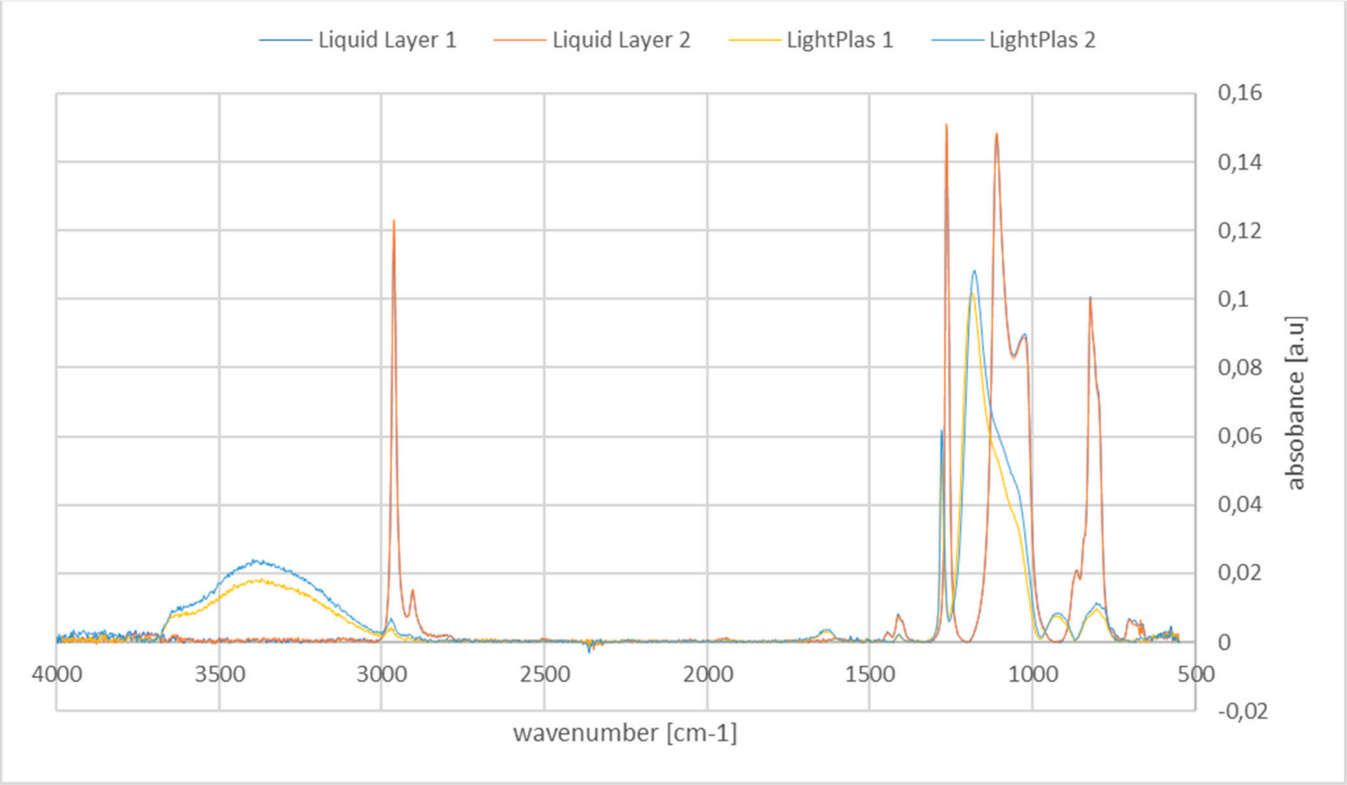

Supplementary Figure S1: IRRAS-spectroscopy for analysis of the chemical composition. Spectra of the liquid silicone layer (AK50) after homogenisation (step three) in comparison to the final LightPLAS coating, i.e., after VUV cross-linking and grafting. The spectra of two individual measurements are shown in each case.
